# Brimonidine as a possible treatment for myopia

**DOI:** 10.1186/s12886-024-03433-6

**Published:** 2024-04-11

**Authors:** Zixuan Peng, Aiqun Xiang, Hong He, Yaqi Luo, Shunliang Wu, Yanting Luo, Junming Yang, Ke Nie, Xingwu Zhong

**Affiliations:** 1 Hainan Provincial Key Laboratory of Ophthalmology, Hainan Eye Hospital, Zhongshan Ophthalmic Center, Sun Yat-sen University, No. 19 Xiuhua Road, Xiuying District, 570300 Haikou, Hainan China; 2 State Key Laboratory of Ophthalmology, Zhongshan Ophthalmic Center, Sun Yat-sen University, Guangzhou, China; 3 Hainan Medical University, Haikou, Hainan China

**Keywords:** Myopia, Guinea pig, Brimonidine, Form deprivation

## Abstract

**Background:**

Myopia is becoming a huge burden on the world’s public health systems. The purpose of this study was to explore the effect of brimonidine in the treatment of form-deprivation myopia (FDM) and the relationship between intraocular pressure (IOP) and myopia development.

**Methods:**

Monocular form deprivation myopia (FDM) was induced in three-week-old pigmented male guinea pigs. They were treated with 3 different methods of brimonidine administration (eye drops, and subconjunctival or intravitreal injections). Four different concentrations of brimonidine were tested for each method (2*µ*g/*µ*L, 4*µ*g/*µ*L, 20*µ*g/*µ*L, and 40*µ*g/*µ*L). All treatments continued for a period of 21 days. Tonometry, retinoscopy, and A-scan ultrasonography were used to monitor intraocular pressure, refractive error and axial length (AL), respectively.

**Results:**

Treatment with subconjunctival brimonidine at 40*µ*g/*µ*L, and intravitreal brimonidine at 2*µ*g/*µ*L and 4*µ*g/*µ*L, inhibited the development of FDM. The myopic refraction, excessive axial length, and elevation of IOP were significantly decreased. Brimonidine in eye drops was ineffective.

**Conclusion:**

Brimonidine at appropriate doses significantly reduced the development of FD myopia in guinea pigs. The IOP may change with FD myopia.

## Introduction

Myopia is a common refractive error, the pathogenesis of which is not fully understood and is mostly associated with excessive growth of the eye axis. In recent years, the prevalence of myopia has gradually increased and has become an increasingly serious public health problem [1], with studies suggesting that by 2050, nearly 5 billion people (50% of the world’s population) will suffer from myopia [2]. If left unattended, myopia will be a huge burden on the public health system [3], so preventing the development of myopia is urgent.

At present, the main drug used to control myopia is atropine. Numerous studies have shown that low concentrations of atropine can slow the progression of myopia in children, and this drug has been approved for clinical use in some countries. However, the exact mechanism by which atropine inhibits myopia remains unclear, and it is associated with certain side effects. Some other drugs have been reported to similarly inhibit myopia progression. Some studies in recent years have shown that brimonidine inhibits the development of myopia in animals. 0.1% and 0.2% brimonidine eye drops effectively inhibited the progression of lens-induced myopia (LIM) in guinea pigs [4]. Intravitreal injection of brimonidine at 20nmol/20µL and 200nmol/20µL was effective in slowing the progression of form-deprivation myopia (FDM) in chicks [5]. 

Brimonidine is a highly selective α2-adrenoceptor agonist that effectively decrease IOP in glaucoma patients by inhibiting aqueous humor production and promoting aqueous outflow. It is a relatively new ocular hypotensive medication [6]. Carr et al. have shown that certain muscarinic acetylcholine receptors (mAChRs) antagonists bind to α2A-adrenergic receptor at high concentrations, leading them to propose α-adrenergic receptors as potential targets for novel myopia intervention strategies [7]. Liu et al. suggest that myopic mammals exhibit a condition known as scleral creep, whereby the sclera becomes more compliant and susceptible to deformation in response to myopiagenic stimuli, promoting AL elongation under IOP. Following treatment with brimonidine, it enhances the expression of neurotrophic factors such as basic fibroblast growth factor (bFGF), and thus affect the remodeling of the sclera. It also leads to a decrease in IOP, and the combined effects inhibit FDM in guinea pigs [4]. 

Based on the above findings, our laboratory discovered that intravitreal injection at 4 µg/µL was effective in slowing the progression of form-deprivation myopia in guinea pigs [8], while the same concentration of brimonidine administered through eye drops and subconjunctival injections proved ineffective. This finding encourages further investigation, as different drug delivery methods affect therapeutic efficacy by influencing drug utilisation. Brimonidine may exert its inhibitory effect on form-deprived myopia (FDM) by stimulating a specific target within the eye, possibly the retina. However, due to factors such as drug evaporation on the ocular surface, rapid clearance via the tear fluid and the presence of barriers including the corneal and conjunctival epithelium and the blood-ocular barrier, the bioavailability of the drug via eye drop and subconjunctival routes of administration is relatively low [9], ultimately resulting in the inability of the drug to reach the target site at effective concentrations. The need of repeated eye puncture with intravitreal injections causes several side effects such as endophthalmitis, hemorrhage, retinal detachment and poor patient tolerance [10]. The other two methods of drug delivery are less invasive and more promising for clinical translation. We hypothesise that higher concentrations of brimonidine may also be effective with these two routes of administration. Therefore, the aim of the present study was to further investigate the effects of different concentrations of brimonidine on myopia in guinea pigs using three modes of administration: eye drops, subconjunctival injection and intravitreal injection, and to investigate the correlation between IOP and myopia and, if such a correlation exists, to establish the causal relationship between the two.

## Materials and methods

### Animals and ethics statement

Three-week-old male tricolored guinea pigs were purchased from Changsha Tianqin Biotechnology Corporation and housed under the following conditions: temperature of 24 °C, daily light: 12:12 h of darkness, adequate food, water and fresh vegetables. All experimental procedures were in accordance with the ARVO Declaration on the Use of Animals in Ophthalmic Research and animal use protocols were approved by the Sun Yat-sen University Institutional Animal Care. The duration of the experiments was 21 days. At the end of the experiment, all animals were sacrificed by intraperitoneal injection of an overdose of pentobarbital sodium(150 mg/Kg) and their eyeballs were removed to be used as control material for other experiments. To ensure the welfare of the animals, our method of euthanasia followed the AVMA Guidelines for the Euthanasia of Animals.

### Experimental groups

One hundred and five three-week-old male pigmented guinea pigs were randomly divided into 17 groups (Table 1): All guinea pigs intervened in the right eye only, the left eye was left untreated.

**Table 1:** Groups

Group	Treatment	Numbers
A	None	6
B	FDM Only	6
C1	FDM+2*µ*g/*µ*L brimonidine eyedrops, one drop	6
C2	FDM+4*µ*g/*µ*L brimonidine eyedrops, one drop	6
C3	FDM+20*µ*g/*µ*L brimonidine eyedrops, one drop	6
C4	FDM+40*µ*g/*µ*L brimonidine eyedrops, one drop	6
C5	FDM+PBS, one drop	6
D1	FDM+2*µ*g/*µ*L brimonidine subconjunctivally, 5*µ*L	6
D2	FDM+4*µ*g/*µ*L brimonidine subconjunctivally, 5*µ*L	6
D3	FDM+20*µ*g/*µ*L brimonidine subconjunctivally,5*µ*L	7
D4	FDM+40*µ*g/*µ*L brimonidine subconjunctivally, 5*µ*L	6
D5	FDM+PBS, 5*µ*L	6
E1	FDM+2*µ*g/*µ*L brimonidine intravitreally, 5*µ*L	7
E2	FDM+4*µ*g/*µ*L brimonidine intravitreally, 5*µ*L	7
E3	FDM+20*µ*g/*µ*L brimonidine intravitreally, 5*µ*L	6
E4	FDM+40*µ*g/*µ*L brimonidine intravitreally, 5*µ*L	6
E5	FDM+PBS, 5*µ*L	6

### Rationale for control eye selection

In this study, we investigated the AL, SE and IOP in guinea pig eyes during the development and treatment of FDM. To induce FDM, we used a black latex balloon formed into a mask to cover the right eye of the guinea pig. However, due to the potential for interaction between the eyes, when comparing AL and SE, we chose to compare group B (FDM only) with the right eyes of the other groups, rather than the left eye of each guinea pig. This decision was based on Several studies have shown that in the same species (e.g., rhesus monkeys [11], mice [12], chicks [13], and guinea pigs [14]), refraction and AL change in the untreated contralateral eye with FDM on the opposite side. By comparing these groups with group B, we aimed to ensure the accuracy of the comparison. Only when comparing intraocular pressure, left eye pressures were used as references.

### Induction of FDM and schedule of brimonidine treatments and refractions

A black latex balloon was made into a mask for the right eye, and the left eye was left uncovered. Eye drops: 1 drop was applied to the right eye at both 9:00 am and 4:00 pm daily. Subconjunctival injection: 5 *µ*L was injected every 4 days into the subconjunctival space. Intravitreal injection: 5 *µ*L was injected every 4 days into the vitreous cavity. All the above interventions (FDM, Eye drops, Subconjunctival injection and Intravitreal injection) were synchronized at the beginning of the experiment and maintained throughout the entire experimental period.

### Measurement of spherical equivalent refraction (SE)

Streak retinoscopy was performed on guinea pigs at baseline and on both day 10 and day 20. Before examination, pupillary dilation and cycloplegia were induced with 1% tropicamide phenylephrine ophthalmic solution (Saten, Osaka, Japan)– administered 3 times, one drop each time, once every 5 min. Twenty minutes after the third drop, in a dark room, an assistant gently held the animal while an experienced optometrist (the same one all the time) performed streak retinoscopy along both axes and recorded the mean of the two measurements as the spherical equivalent (SE) refraction.

### Measurement of ocular axial dimensions

A-scan ultrasound with a 25-MHz probe (AXIS-II; Quantel Medical Inc., Clermont- Ferrand, France) as performed on guinea pigs at baseline and on days 7, 14, and 21. The right eye was anesthetized with proparacaine hydrochloride drops and the guinea pig was restrained manually. The examiner placed the ultrasound probe on the corneal surface at the center of the pupil and recorded the values. For each measurement, at least five traces were captured per eye and analysed offline. All measurements were done by the same skilled examiner.

### Measurement of intraocular pressure

The right eyes of calm animals were anesthetized topically with 0.5% proparacaine hydrochloride (s.a. ALCON-COUVREUR n.v.), and a rebound IOP meter (Solvay SW-500) was used. An assistant restrained the animal by hand, the examiner aimed the probe vertically at the central cornea, IOP was measured rapidly five times in each eye, and the average value was calculated and recorded. At the end of the examination, levofloxacin drops were administered to the eyes to prevent infection. All intraocular pressure measurements were conducted in awake animals prior to procedures such as subconjunctival or intravitreal injections, in order to avoid potential confounding effects of the latter and all examinations were done by the same skilled operator.

### Statistical analysis

The statistical software used was SPSS22.0, and data were expressed as mean *±* standard error. If the homogeneity test of variance was satisfied, two-way repeated measures ANOVA with a Bonferroni post hoc test was applied to longitudinal data. If the data did not meet the homogeneity test of variance, the Kruskal-Wallis test was used; *P* < 0.05 was taken as statistically significant.

## Results

### Axial length

**Fig. 1:**
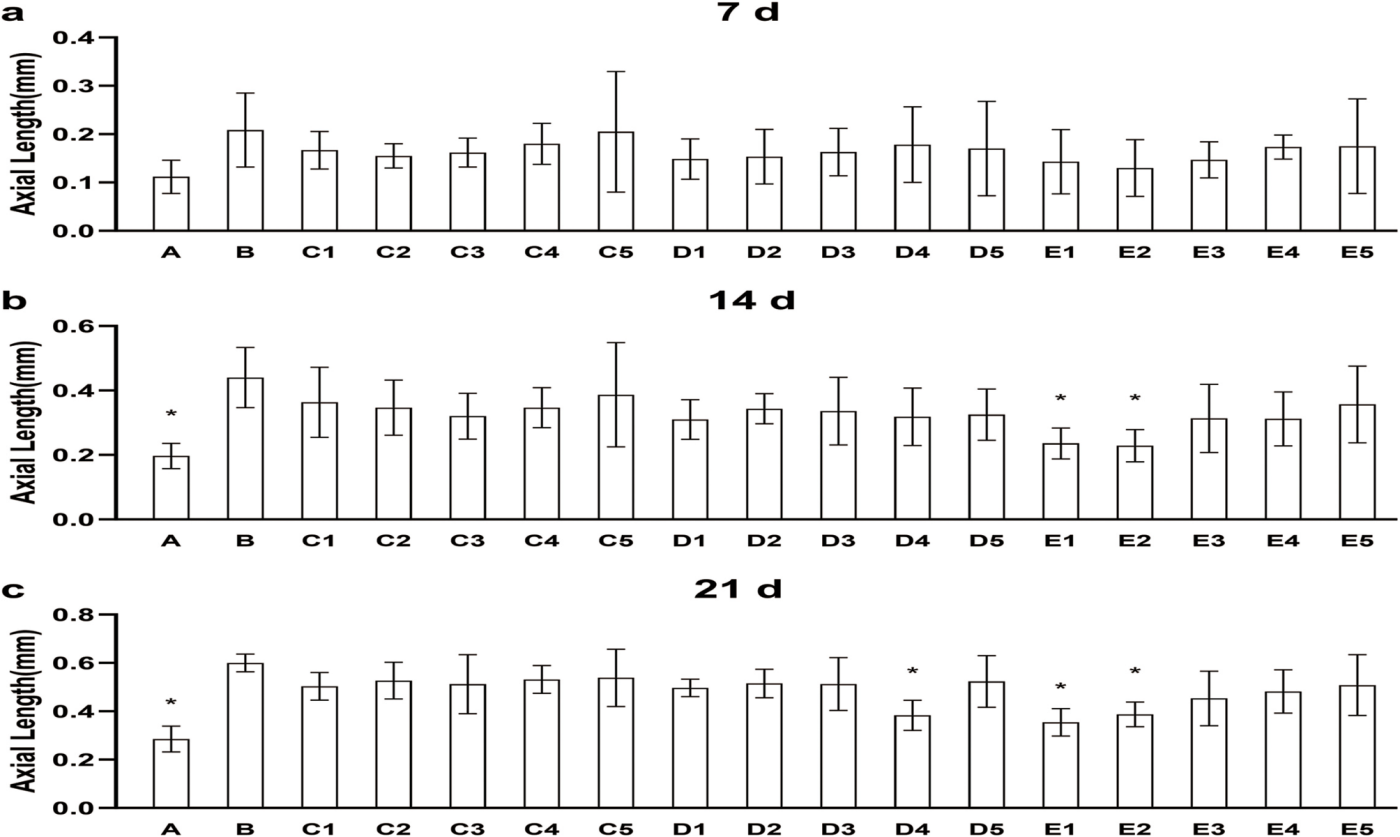
(a) Change in AL (7d– 0d) over the course of 7 days. (b) Change in AL (14d– 0d) over the course of 14 days. (c) Change in AL (21d– 0d) over the course of 21 days. *P < 0.05vs B. A: No treatment; B: Monocular FD alone; C: FD + eyedrops; D4: FD + subconjunctival; E: FD + intravitreal

**Fig. 2:**
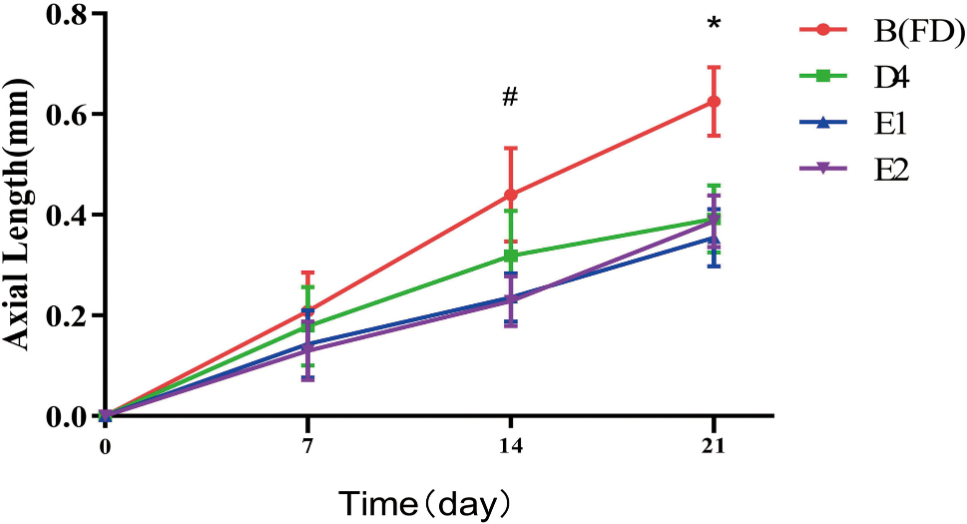
Change in AL over the course of 3 weeks. B: Monocular FD, no drug; D4, and E1,2: FD + brimonidine at stated concentration, by different delivery routes. D4: FD + 40*µ*g/*µ*L subconjunctival; E1,2: FD + 2.0*µ*g/*µ*L, 4.0*µ*g/*µ*L intravitreal. *P < 0.05 B compared to values for all other groups. #*P* < 0.05 B compared to values of the other groups, excluding D4

As shown in Figs. 1 and 2, the change in axial length (AL) difference value after treatment for 7, 14, and 21 days, between groups E1(2.0 µg/µL, intravitreal), E2(4.0 µg/µL, intravitreal) and group B, were statistically significant (*P* < 0.05), from day 14 onward. The trend of increases with time was similar in the two treatment groups at day 14. There was a statistically significant difference between the D4(40 µg/µL, subconjunctival) group and the B group on day 21. The magnitude of change was smaller in these three groups than in group B.

It is important to note that brimonidine inhibited excessive axial elongation in FD eyes, only at some doses and only when administered intravitreally or subconjunctivally. Brimonidine had no inhibitory effect on AL when administered in eye drops, at any dose test.

### Spherical equivalent

**Fig. 3:**
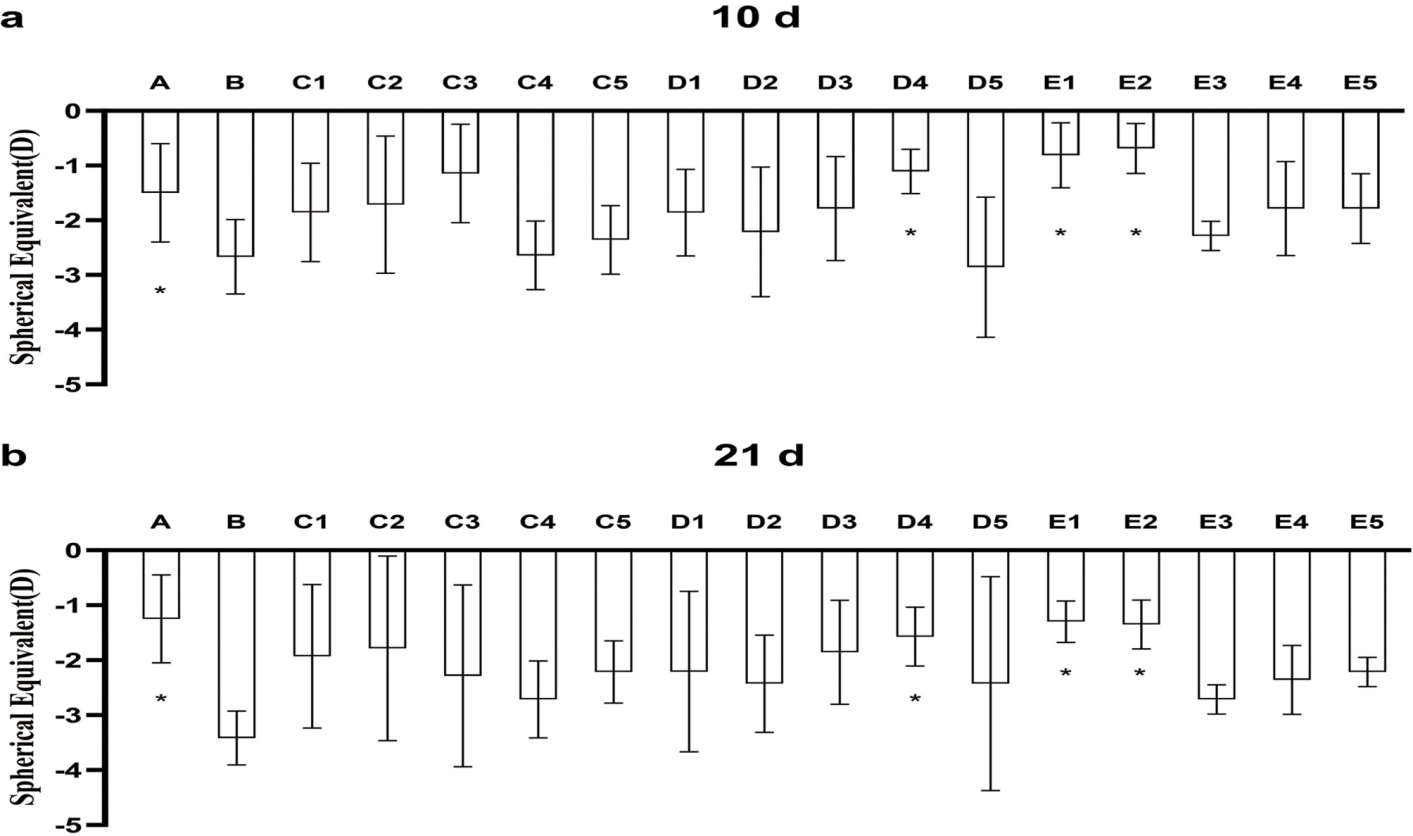
(a) Change in SE refractive error (10d– 0d) over the course of 10 days. (b) Change in SE refractive error (21d -0d) over the course of 21 days. *P < 0.05 vs. B

**Fig. 4:**
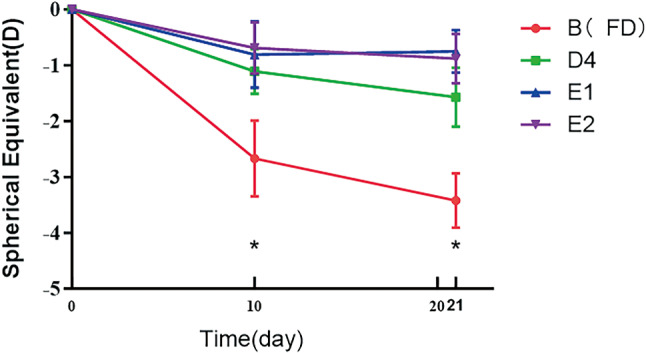
Change in SE refractive error over the course of 3 weeks. *P < 0.05 B compared to values for all other groups

As seen in Figs. 3 and 4, the change in spherical equivalent (SE) of refractive error difference, after 10 days and 21 days of treatment, were statistically significant. Furthermore, pairwise comparison showed that the differences between data for group B and those for each of the other three groups were statistically significant.

As in the case of axial length, brimonidine inhibited excessive myopic shift in refraction in FD eyes, only at some doses and only when administered intravitreally or subconjunctivally. It had no inhibitory effect when administered in eye drops, at any dose tested.

### Comparison of IOP in each group

**Fig. 5:**
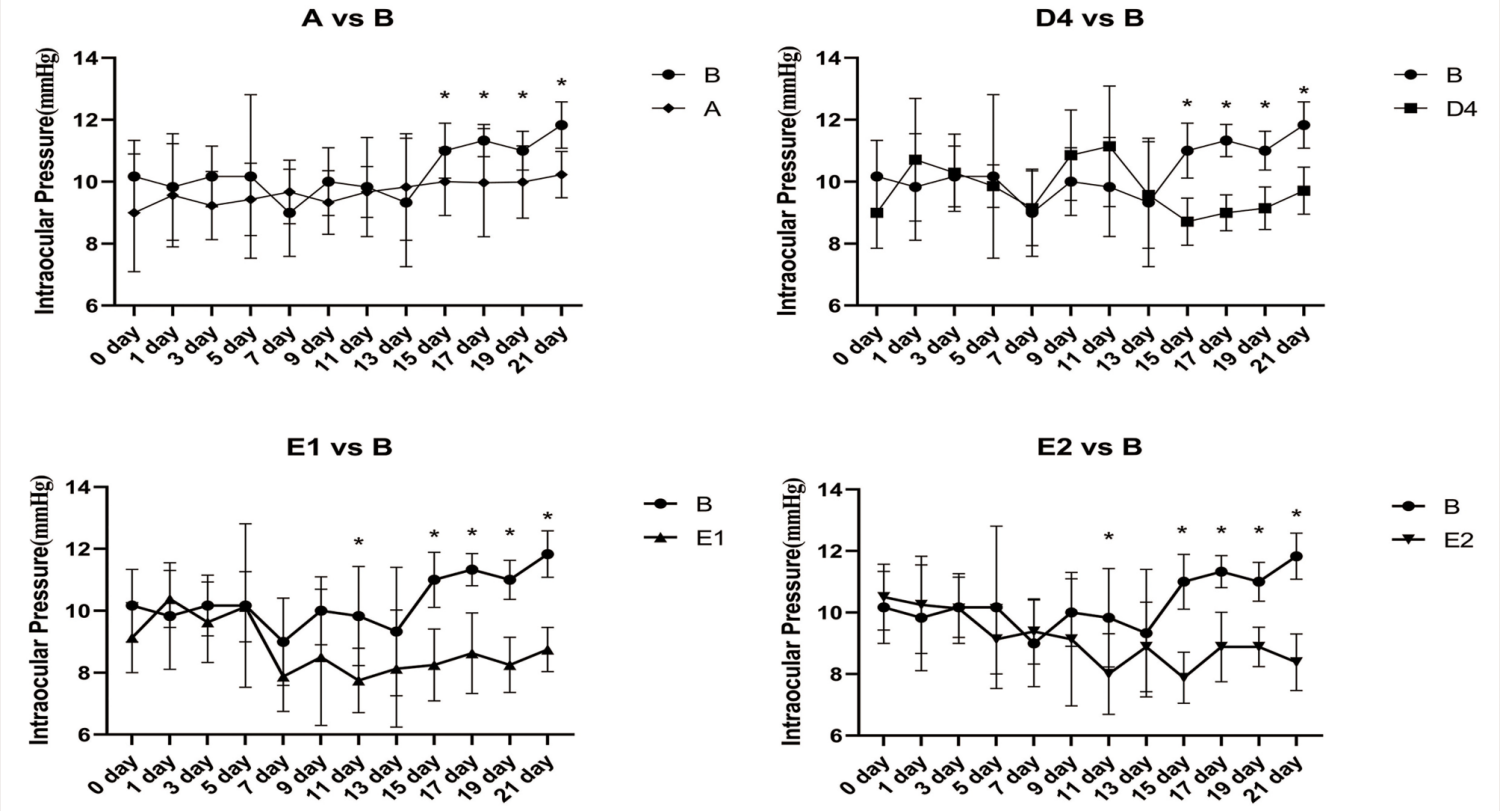
Changes in intraocular pressure over the course of 3 weeks. *P < 0.05 vs. B

As can be seen from and Fig. 5, the change in intraocular pressure (IOP) between groups at 3, 7, 9, 11, 15, 17, 19, and 21 days were statistically significant. Further pairwise comparisons showed that the inter-group differences in IOP were statistically significant for all four treatment groups compared with B, at 15, 17, 19, and 21 days.

### Correlation analysis of IOP and AL

Correlation analysis between AL difference values and IOP in groups B, D4, E1 and E2, showed that they were positively correlated in all 4 groups. This shows that as interocular differences in AL become larger, so also do those differences in IOP. The correlation between AL difference values and IOP was highest in group E1 (*r* = 0.925, *P* < 0.001) and lower in groups B (*r* = 0.899, *P* < 0.05), D4 (*r* = 0.773, *P* < 0.05) and E2 (*r* = 0.724, *P* < 0.05).

## Discussion

### Effective drug delivery method and concentration

Brimonidine has been shown to be effective at inhibiting the development of myopia in animal models. Carr et al. found that brimonidine at 20nmol/20*µ*L (approximately 0.442ug/*µ*L) and 200nmol/20*µ*L (approximately 4.422ug/*µ*L) of injected intravitreally was effective in slowing the progression of form-deprivation myopia in chicks [5]. Our groups previously reported similar results, in that that intravitreal injection of 4*µ*g/*µ*L brimonidine was effective in slowing the progression of form-deprivation myopia in guinea pigs [8]. The results of the present experiment, specifically that 4ug/*µ*L intravitreally is effective, are generally consistent with the results of these two studies. In contrast to Liu et al. who found that brimonidine eye drops inhibited the progression of lens-induced myopia (LIM) in guinea pigs, in the present study we found that brimonidine eye drops did not slow the progression of form-deprivation myopia (FDM) in guinea pigs [4]. Differences in outcomes of these studies might be due to differences in (e.g.) strains of guinea pigs, ways of delivering eye drops, instrumentation, and techniques for measuring SE and AL; but the most likely explanation lies in the different ways of inducing myopia, as some research findings have suggested different mechanisms for LIM and FDM [15, 16]. 

We also found that subconjunctival injection of 40ug/*µ*L and intravitreal injection of 2ug/*µ*L brimonidine were effective in suppressing myopia development, while intravitreal injections of higher concentrations (20ug/*µ*L and 40ug/*µ*L) were ineffective. It might be due to the off-target binding of high concentrations drugs. We speculate that brimonidine– like many other ligands - is not specific to a single kind of *α*2-adrenoceptor or other receptors but becomes active at other receptor(s) at higher concentrations; similarly, biphasic responses to drugs over a wide range of concentrations have been reported for the effects of dopaminergic agents on naturally occurring myopia in albino guinea pigs [17]. In the same way, the three mAChR antagonists— atropine, himbacine, and MT3-bind to human α2A-adrenoceptors when administered at or above concentrations of 45µmol/L, 17 µmol/L, and 15 nmol/L, respectively, in HEK293T cells [7]. The subconjunctival injection of 40ug/*µ*L of brimonidine could reach the same concentration as the vitreous injection of 2ug/*µ*L and 4ug/*µ*L, in the retina or other target tissues, and thus inhibit the effect of FDM. We have been unable to find any published studies on the pharmacokinetics and cell or tissue targets of brimonidine after subconjunctival or intravitreal injections, and further studies will be needed to understand the bases of such anomalous responses to the drug.

### Exploration of the causal relationship and mechanism of IOP and ocular axis

Research on the correlation between IOP and myopia has been a hot topic of study in recent years; however, the exact relationship between IOP and myopia is controversial. An early human study found that after excluding factors such as amblyopia, strabismus, prematurity, age, and family history of myopia, myopia was still strongly correlated with IOP [18]. Several similar studies have shown that IOP was significantly higher in the high myopia group than in the control group and that it was significantly correlated with ocular axial length [19–21]. However, contrary to the findings in those reports, two other studies found no statistically significant differences in IOP between control and myopia groups or between groups with different degrees of myopia [22, 23]. 

**Fig. 6:**
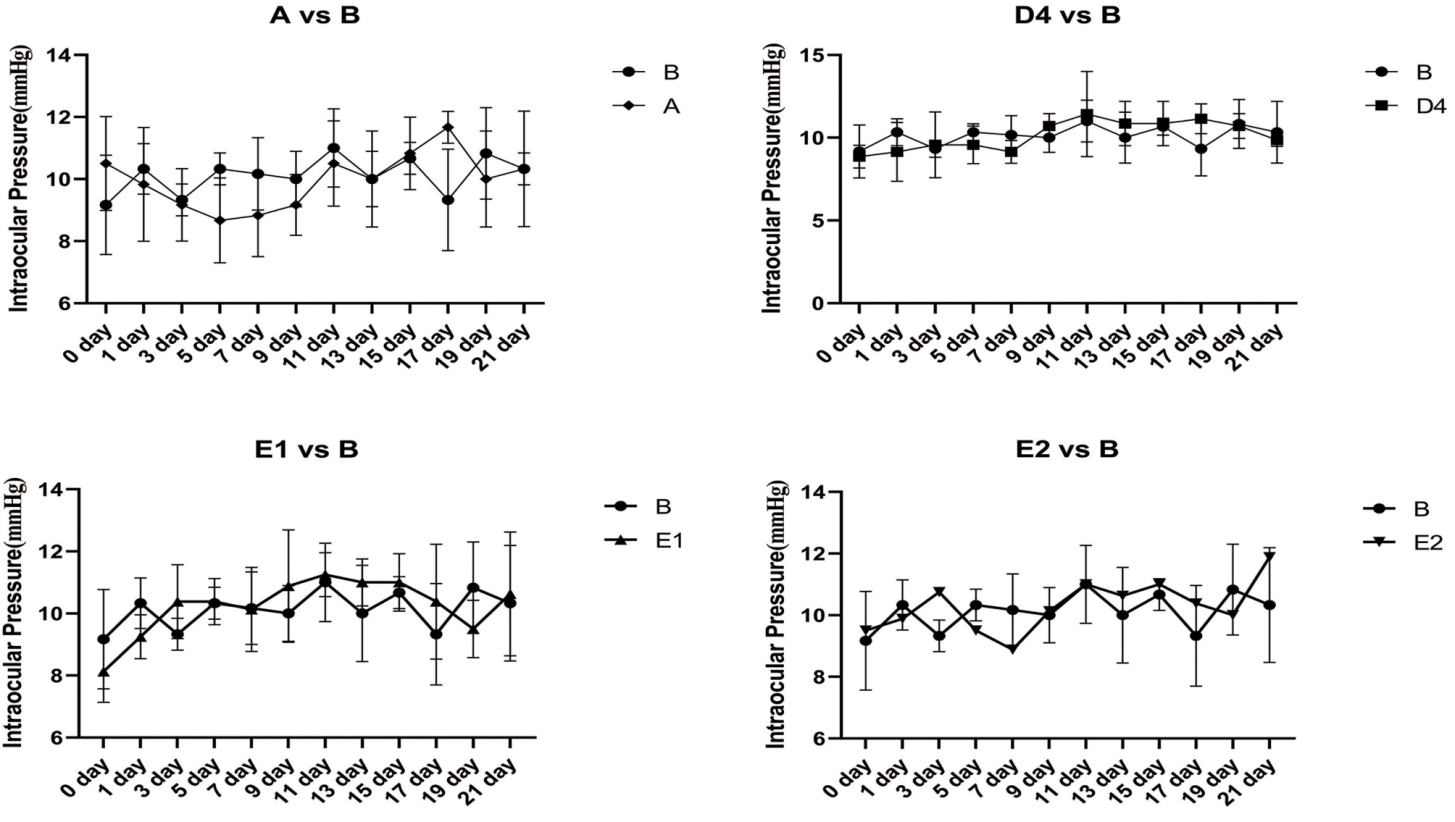
Correlations of AL with IOP in baseline group B and treatment groups D4, E1 and E2

**Fig. 7:**
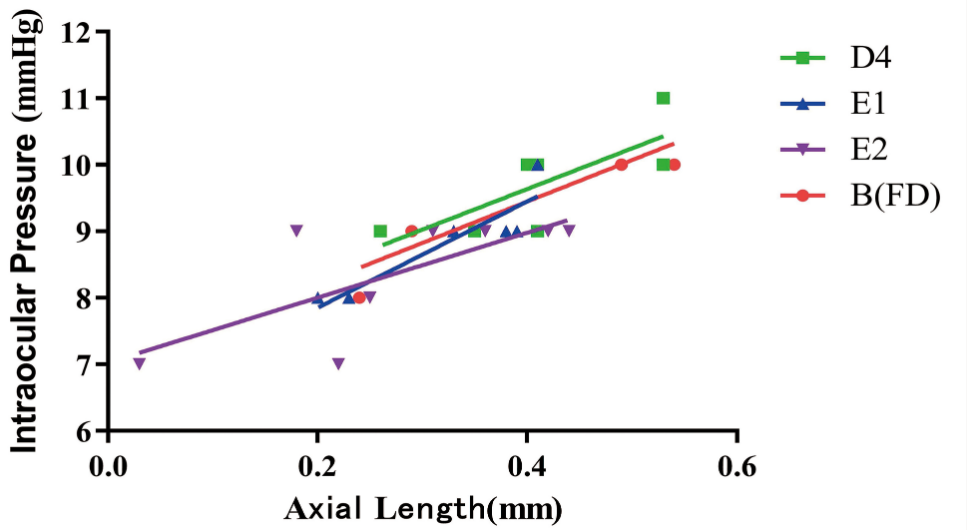
Changes in intraocular pressure over the course of 3 weeks in left eye (untreated eye)

Comparing our multiple group data by ANOVA revealed that the IOPs in groups D4, E1, and E2 were significantly lower than those in group B, from day 14 onwards (Fig. 5). Correlation analysis of data for groups B, D4, E1, and E2 showed that all correlations were positive (Fig. 6), with IOP and AL tending to increase in the simple form deprivation group (group B) and to decrease in the brimonidine-responsive group (D4, E1, and E2), compared to group B. But the left eyes (untreated eye) of these 3 groups were essentially unchanged compared to group B (Fig. 7); these findings in the present study are consistent with the results of previous studies [8]. IOP was highly correlated with AL in group E1 (*r* = 0.925, *P* < 0.001), and less highly but still significantly correlated with AL in the B, D4, and E2 groups (*r* = 0.899, *P* < 0.05 in group A; *r* = 0.773, *P* < 0.05 in group D4; and *r* = 0.724, *P* < 0.05 in group E2). It remains unclear whether there is a causal relationship between IOP and AL, and it has been suggested [24] that lowering IOP inhibits the activation of scleral fibroblasts, thereby reducing scleral remodelling, and that a decrease in scleral dilatation force retards the balloon-like expansion of the scleral coat. It has also been suggested that lowering IOP leads to increased choroidal blood perfusion, which reduces scleral hypoxia and is accompanied by decreases in scleral remodelling. However, there is no conclusive evidence that high IOP causes myopia, and on the contrary, there is convincing evidence that scleral enlargement (stretching or active growth), rather than increased IOP, is most important. For example, in chick as well as several mammalian models it has been shown that hemiretinal form deprivation or hyperopic defocus produced localized axial elongation– specifically, the form-deprived or imposed-defocus region became larger, whereas the untreated regions of the eye wall did not [25–27]. Since pressure in a fluid is exerted equally in all directions, and if high IOP causes myopia by affecting scleral compliance, then the vitreous chamber should enlarge uniformly, not locally. Based on the results of the current experiment, with regard to group B (Monocular FD alone), the guinea pigs in this group did not receive brimonidine treatment, it is observed that group B began to show a decrease in IOP on day 15 (Fig. 5), while the spherical equivalent (SE) began to show myopia on day 10 (Fig. 3). The change in IOP lagged behind the changes in SE; therefore, the elevated IOP could simply be a consequence of myopia production.

Most studies suggest that the control of myopia progression is primarily mediated through the action of mAChRs [28, 29], However, recent studies have shown that atropine is unlikely to inhibit myopia progression by acting upon mAChRs, and more likely does so via adrenergic receptors [30]. For example, McBrien et al. found in myopia experiments with chicks and tree shrews that retinal acetylcholine (ACh) levels in myopic animals were not significantly different from those in controls [31]. In addition, many other antagonists of mAChRs, unlike atropine, did not inhibit the development of myopia [32]. Thomson et al. also found that muscarinic, nicotinic, and non-specific cholinergic agonists inhibited FDM development, leading them to question whether atropine inhibits myopia via cholinergic antagonism [33]. These findings suggest that mAChRs may not affect the development of myopia. In addition, Näreoja found that certain muscarinic toxins not only interfere with binding of acetylcholine to its receptors, but also have moderate to high affinity for adrenergic receptors [34]. It has even been found that atropine interacts with α-adrenoceptors in addition to muscarinic receptors [35]. Carr’s group also found that muscarinic antagonists block signaling via α2A-adrenoceptors at concentrations comparable to those used to inhibit chick myopia in vivo [7]. Currently, some experimental animal studies have found that α2-adrenoceptor agonists can inhibit the development of myopia in animals (including chicks and guinea pigs) [4, 5, 8]. The results of these studies suggest that α2-adrenergic receptors may be the real target of atropine’s action in inhibiting myopia development. However, while brimonidine is equally effective as atropine in inhibiting form-deprivation myopia in chicks [5, 32], the required concentration still greatly exceeds the effective concentrations found in receptor binding and activity assays [36, 37]. Another study published by our experimental group [8] also found that the expression of adrenergic signaling-related genes in the retina of form-deprived guinea pig eyes injected with brimonidine was not significantly different from that of the control group. This might suggest that the mechanism by which brimonidine slows myopia progression is not mediated by adrenergic signaling-related pathways. The specific mechanism by which brimonidine slows myopia progression still needs to be investigated further.

Although the guinea pig eye is similar to the human eye in many ways, if we want to study it further in monkeys or humans, we need to know the differences between the guinea pig eye and the primate eye. For example, the guinea pig retina is avascular and under physiological conditions the oxygen content of the retina and choroid is much lower than in other animals with vascularised retinas [38]. Pharmacokinetic experiments are required to determine the efficiency of retinal absorption rates. Compared to primates, guinea pigs are more hyperopic at birth and undergo emmetropization during post-natal development. Emmetropization in guinea pigs is rapid during the first 3 weeks of age and then slows down. During the first five weeks after birth. the increase in axial length in guinea pigs is primarily determined by the thickening of the crystalline lens. However, the axial growth of the eye in primates is mainly determined by lengthening of the vitreous chamber, followed by deepening of the anterior chamber and the lens thickening [39]. This suggests that the timing of brimonidine administration in relation to these developmental processes may require further discussion.

Despite these results showing the inhibitory effect of brimonidine on myopia, this study has limitations. Firstly, this experiment did not investigate the mechanisms and pathways involved. The mechanism leading to the occurrence of myopia has been widely investigated, and factors such as retinal dopamine secretion [40], scleral extracellular matrix [41, 42], and scleral hypoxia [43] have been implicated, but none of them can fully explain the mechanism of myopia in this study. Secondly, due to budgetary constraints, the experimental animals used in this study were guinea pigs. Although guinea pigs, like humans, are mammals, they differ to some extent from humans in terms of eye structure and the aetiology of myopia. This is not conducive to further clinical trials of this drug. If future researchers are interested in further studies, it is suggested that primates, which are closer to humans, should be used as research subjects.

In conclusion, our study suggests brimonidine at appropriate doses significantly reduced the development of FD myopia in guinea pigs. The IOP may change with FD myopia. There is a positive correlation between IOP and AL, with IOP increasing as AL increases. Brimonidine is a highly promising drug for future myopia treatment.

## Data Availability

Data can be made available from the corresponding author at 32841542@qq.com on a reasonable request.
